# The association between exposure to different aspects of shift work and metabolic risk factors in health care workers, and the role of chronotype

**DOI:** 10.1371/journal.pone.0211557

**Published:** 2019-02-01

**Authors:** Bette Loef, Debbie van Baarle, Allard J. van der Beek, Piet K. Beekhof, Linda W. van Kerkhof, Karin I. Proper

**Affiliations:** 1 Center for Nutrition, Prevention and Health Services, National Institute for Public Health and the Environment, Bilthoven, The Netherlands; 2 Department of Public and Occupational Health, Amsterdam Public Health research institute, VU University Medical Center, Amsterdam, The Netherlands; 3 Center for Infectious Diseases Control, National Institute for Public Health and the Environment, Bilthoven, The Netherlands; 4 Department of Immunology, Laboratory for Translational Immunology, University Medical Center, Utrecht, The Netherlands; 5 Center for Health Protection, National Institute for Public Health and the Environment, Bilthoven, The Netherlands; University of Lausanne, SWITZERLAND

## Abstract

**Objective:**

Shift work has been linked to cardio-metabolic diseases, but insight into different shift work-related aspects and chronotype of shift workers and their relation with metabolic risk factors is limited. This study examined the association between current shift work status, frequency and duration of night shift work, chronotype, and metabolic risk factors in a population of health care workers.

**Methods:**

Anthropometrics, questionnaires, and blood samples were collected from 503 shift working and 93 non-shift working health care workers employed in hospitals. Body mass index, waist circumference, cholesterol (total, HDL, LDL), triglycerides, and high-sensitivity C-reactive protein were measured. Associations of current shift work, frequency (non-night shift worker, 1–2, 3–4, ≥5 night shifts/month) and duration of night shift work (non-night shift workers, <10, 10–19, ≥20 years), and shift workers’ chronotype, with metabolic risk factors were studied using linear regression analysis.

**Results:**

Compared to non-shift workers, shift workers’ total cholesterol level was 0.38 mmol/L lower (95%-CI = -0.73 –-0.04) and LDL cholesterol was 0.34 mmol/L lower (95%-CI = -0.60 –-0.08). For all other metabolic risk factors, no differences were found. The association between shift work and LDL cholesterol was especially found among shift workers working night shifts for ≥20 years (B = -0.49 (95%-CI = -0.78 –-0.19)). No differences were found for night shift frequency and chronotype.

**Conclusion:**

In this population of health care workers employed in hospitals, no evidence for differences in metabolic risk factors was observed that could underlie a link between shift work and cardio-metabolic diseases. Further research using different aspects of shift work to study the association with metabolic risk factors is recommended.

## Introduction

In today’s society, working night shifts and other shifts outside normal working hours is an integral part of the jobs of many workers. As shift work will likely continue to exist in the future, it is important to study possible health consequences of shift work.[1] In recent years, researchers have gathered growing evidence linking shift work to overweight and cardio-metabolic diseases, such as cardiovascular diseases and diabetes mellitus type 2.[2–5] For example, two systematic reviews of longitudinal studies found evidence for a relation between shift work and body weight gain.[3, 4] Furthermore, other systematic reviews and meta-analyses suggest that shift work is associated with an increased risk of metabolic syndrome [6], cardiovascular diseases [5, 7], and diabetes mellitus type 2.[2] As overweight, cardiovascular diseases, and diabetes mellitus type 2 are highly prevalent diseases that are associated with increased mortality and morbidity, insight into the influence of shift work on these health problems is of great public health importance.

Although shift work has been linked to the onset of cardio-metabolic diseases, little is known about the association between shift work and important risk factors of these diseases. Yet, studying these risk factors could contribute to understanding the underlying mechanisms of the negative health effects of shift work. In addition, gaining insight into the association between shift work and metabolic risk factors, such as body mass index (BMI) [8], waist circumference [9], cholesterol (total, high-density lipoprotein (HDL), low-density lipoprotein (LDL)) [9, 10], triglycerides [10], and high-sensitivity C-reactive protein (HS-CRP) [11, 12], is useful from a secondary prevention view, because the actual disease may not have been established yet. Previous reviews to such metabolic risk factors showed insufficient evidence for a link between shift work and specific metabolic risk factors (e.g. lipid metabolism).[3, 4, 13] Main reasons for this insufficient evidence were inconsistencies between the studies and lack of high quality research.

An important shortcoming, so far, is the lack of a comprehensive assessment of shift work. Information about frequency and total duration of (night) shift work may provide better insight into the adverse cardio-metabolic health effects of (long-term) shift work exposure, and could help to identify high risk groups for negative health effects of shift work. Next to shift work-related aspects, individual characteristics may also increase a person’s risk for adverse health effects of shift work.[14] For example, chronotype, i.e. an individual’s internal timing of waking and sleeping, has been shown to play a role in the effect of shift work on health.[15] To date, multiple studies have reported that morning types may be less able to adapt to shift work than evening types.[1, 14, 15] However, research into the role of chronotype in the effect of shift work on metabolic risk factors is still lacking.[16] For instance, the question of whether shift workers who are morning types also experience greater disruption of metabolic risk factors compared to shift workers who are evening types remains unanswered.

The aim of the current study was to study the association between exposure to different aspects of shift work (i.e. current shift work status, frequency and duration of night shift work) and metabolic risk factors for cardiovascular diseases and diabetes mellitus type 2, i.e. BMI, waist circumference, total cholesterol, HDL cholesterol, LDL cholesterol, triglycerides, and HS-CRP. Furthermore, it was examined whether these metabolic risk factors were different for shift workers with morning and evening chronotypes.

## Methods

### Study population and design

**Fig 1:**
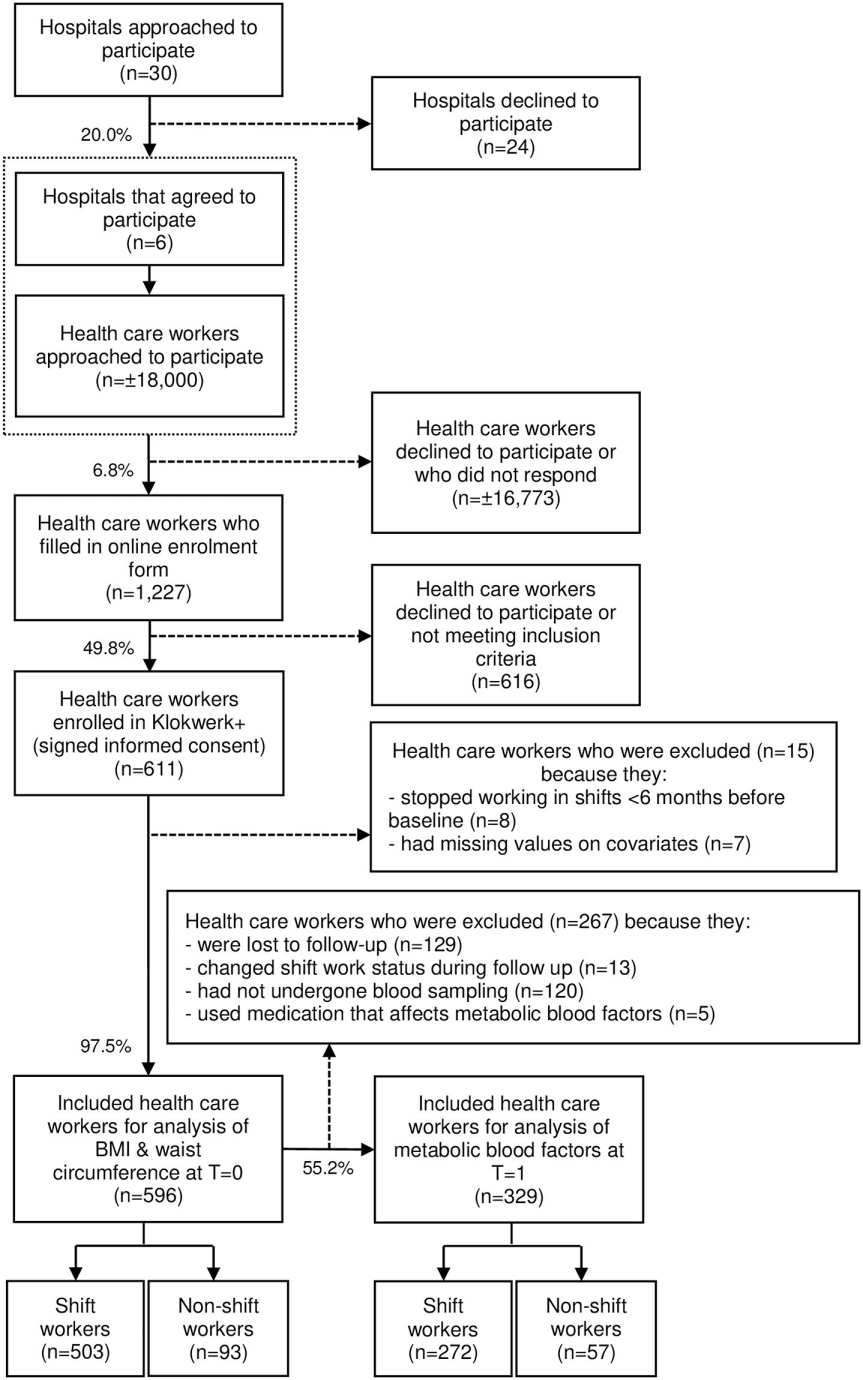
Flowchart of study participants.

The current study is part of Klokwerk+, which is a study exploring the effects of shift work on body weight and infection susceptibility, and the mechanisms underlying these health effects.[17] For this study, 611 health care workers from six different hospitals in the Netherlands were recruited and measured at baseline in the period September-December 2016 (Fig 1). The follow-up measurement took place after approximately six months in the period April-June 2017. Measurements consisted of a questionnaire and measurements of body weight, body height, and waist circumference. At follow-up, blood samples were drawn from a subsample of 347 participants. Data from the anthropometric measurements at baseline and the blood parameters measured at follow-up were used for the analyses. Approval of the current study was obtained from the institutional review board of the University Medical Center Utrecht, Utrecht, The Netherlands on March 15, 2016 (study protocol number 16-044/D, NL56022.041.16). Written informed consent was obtained from all participants.

### Measures

#### Shift work

Information about current shift work status (work schedule and shift types), frequency of (night) shifts (number of each shift type/month), and duration of (night) shift work (start and (if applicable) stop date and total number of years of (night) shift work) was collected at baseline and follow-up. The questionnaire was designed to cover all major aspects of shift work.[18] Participants who worked rotating shifts (rotating between day (mostly between 07.30–16.00), night (mostly between 23.00–07.45), evening (mostly between 15.00–23.00), and/or sleep shifts) and/or night shifts (shifts between 00.00–06.00 am) were considered shift workers. Participants who did not work rotating and night shifts for at least six months were considered non-shift workers. Frequency of night shift work was categorized into 1–2, 3–4, or ≥5 night shifts/month and duration of night shift work was categorized into <10, 10–19, or ≥20 years of night shift work for the night shift workers.

#### Anthropometrics

Participants’ body height (to the nearest 0.5 centimeter using a stadiometer), body weight (to the nearest 0.2 kilogram using a digital weighing scale), and waist circumference (to the nearest 0.1 centimeter using a measuring tape) were measured by the research team following a standardized protocol in which the correct execution of all measurements was systematically described. Measurements took place without shoes and with emptied pockets. Measurements were performed twice, and in the case of a difference of more than one unit (in centimeters/kilograms) between these two measurements, a third measurement was performed. Subsequently, the average of the two values closest to each other was calculated. Body Mass Index (BMI) was calculated by dividing body weight in kilograms by the square of body height in meters.

#### Blood parameters

Blood samples were collected using a standard phlebotomy technique of venipuncture of forearm veins. Total cholesterol, HDL cholesterol, LDL cholesterol, triglycerides, and HS-CRP were measured in non-fasted EDTA-serum. All blood parameters were determined with an auto-analyzer (Unicel DxC 800, Beckman-Coulter, Woerden, the Netherlands), using kits from Beckman-Coulter. The intra-assay variation of the assays was low (total cholesterol 0.81%, HDL cholesterol 0.75%, LDL cholesterol 0.82%, triglycerides 1.19%, HS-CRP 1.65%). Recommended values of the blood parameters are <5.0 mmol/L for total cholesterol, >1.0 mmol/L (males) and >1.3 mmol/L (females) for HDL cholesterol, ≤2.5 mmol/L for LDL cholesterol, <1.7 mmol/L for triglycerides, and <1.0 mg/L for HS-CRP.[9, 10, 19, 20] HS-CRP levels >10 mg/L were excluded (n = 5), because these levels are suggestive of acute infection or other systemic inflammatory process instead of cardiovascular risk.[19]

#### Chronotype

Chronotype was measured with one question in which participants were asked to indicate if they are a morning, evening, or no specific type (intermediate type). This question was based on the Munich ChronoType Questionnaire (MCTQ).[21] Self-assessing one’s chronotype through a single question has been found to give largely the same result as an extended validated questionnaire with questions about preferred times to perform daily activities.[21, 22]

#### Covariates

Age, gender, occupation (nurse vs. other), educational level, marital status, general perceived health (measured using one RAND-36 item about how participants perceive their health, reported on a 5-point Likert scale (excellent—bad)), smoking status (current smoker vs. non-smoker), and alcohol intake (≤7 glasses/week vs. >7 glasses/week) were included as covariates in this study. To gain more insight into the main lifestyle behaviors of the study population, information about physical activity, sleep, and diet was provided. Physical activity level (minutes of moderate to vigorous activity per week during leisure and at work) was measured using the Short Questionnaire to ASses Health enhancing physical activity (SQUASH).[23] Self-reported sleep quality (reported on a scale from very good—very bad) and sleep duration (in hours/day) in the past 4 weeks was measured using one item from the Pittsburgh Sleep Quality Index (PSQI) and one item from the Medical Outcomes Study (MOS) Sleep Scale.[24, 25] Dietary intake was measured with 3-day food dairies based on which meal and snack frequency (in meals/snacks per day) was assessed using the Food Based Classification of Eating episodes (FBCE).[26]

#### Statistical analysis

Descriptive statistics were used to identify the distribution and variation of the main characteristics of the study population.

Linear regression analysis was used to study the association between shift work and the different metabolic risk factors (BMI, waist circumference, total cholesterol, HDL cholesterol, LDL cholesterol, triglycerides, and HS-CRP). For triglycerides and HS-CRP, analyses were performed using the log-transformed values, because of their skewed distribution. A priori, the models were adjusted for age, gender, occupation, and educational level. Further, marital status, general perceived health, smoking status, and alcohol intake were explored as possible confounders by adding them one by one to the models and subsequently checking whether the regression coefficients changed with more than 10%. If so, then the covariate was added to the adjusted model as confounder. Effect modification was examined for age and gender, by adding interaction terms of the covariates and shift work to the adjusted models; interaction was defined as p<0.05. Chronotype was also explored as possible effect modifier, but because of the focus of this study, it was decided a priori to stratify shift workers based on their chronotype in a separate analysis.

All models were repeated with frequency of night shifts (non-night shift workers, 1–2, 3–4, or ≥5 night shifts/month) and duration of night shift work (non-night shift workers, <10, 10–19, or ≥20 years of night shift work) as determinants. Furthermore, analyses were also stratified by chronotype of shift workers (morning, evening, or intermediate type). For all models, the non-shift workers were used as reference group.

Analyses were carried out using IBM SPSS Statistics, V.24.0 (New York: IBM Corp).

## Results

### Study population

Out of the 611 participants included in the Klokwerk+ study, 596 health care workers with complete data were included for the analyses with BMI and waist circumference as outcome (Fig 1). After exclusion of workers who changed their shift work status from baseline to follow-up, who had not undergone blood sampling, and who used medication that affects metabolic blood factors, 329 workers could be included in the analyses of metabolic risk factors measured in blood (Fig 1). Table 1 shows that shift workers were younger (40.9 years vs. 46.8 years, p<0.001), more often nurses (82.7% vs. 33.3%, p<0.001), and less often higher educated (55.1% vs. 74.2%, p = 0.001) than non-shift workers. Furthermore, 41.2% of shift workers were evening types compared to 23.7% of non-shift workers (p = 0.002).

**Table 1:** Characteristics of the study population stratified for non-shift workers and shift workers.

	Shift workers (n = 503) *% or mean (SD); median*	Non-shift workers (n = 93) *% or mean (SD); median*
Age (in years)	40.9* (12.2); 42.0	46.8* (11.2); 49.0
Gender (% female)	88.1	83.9
Occupation (% nurse)	82.7*	33.3*
Educational level (% high)	55.1*	74.2*
Marital status (% married/living together)	73.2	76.3
General perceived health (% very good/excellent)	44.3	37.6
Smoker (% yes)	12.5	5.4
Alcohol intake (% >7 glasses/week)	23.1	17.2
Chronotype		
Morning type (%)	35.8*	50.5*
Evening type (%)	41.2*	23.7*
Intermediate type (%)	23.1	25.8
Physical activity during leisure (in minutes/week)^1^	601.8 (559.8); 450.0	730.5 (699.0); 525.0
Physical activity at work (in minutes/week)^1^	793.8* (637.4); 600.0	325.7* (550.4); 0.0
Sleep duration (in hours/day)^2^	7.3 (0.9); 7.0	7.2 (1.0); 7.1
Sleep quality (% fairly/very good)^2^	80.3*	91.4*
Meal frequency (in number/day)^3^	2.7 (2.0–3.0)	2.3 (2.0–3.0)
Snack frequency (in number/day)^3^	3.3 (2.3–4.0)	3.0 (2.2–4.0)

* Statistically significant difference (p<0.05) between shift workers and non-shift workers tested with independent-samples t-test or chi-square test.

^1^ Based on self-reported data from the Short Questionnaire to ASses Health enhancing physical activity (SQUASH) among 484 shift workers and 91 non-shift workers.

^2^ Based on 501 shift workers and 92 non-shift workers.

^3^ Median (interquartile range). Based on 408 shift workers and 77 non-shift workers.

### Current shift work status and metabolic risk factors

Table 2 compares the values of the metabolic risk factors between shift and non-shift workers. Shift and non-shift workers had similar average values for BMI (25.3 kg/m^2^ vs. 25.3 kg/m^2^, p = 0.865) and waist circumference (85.2 cm vs. 86.4 cm, p = 0.357). However, shift workers had a lower mean level of total cholesterol (5.54 mmol/L vs. 5.99 mmol/L, p = 0.008) and LDL cholesterol (3.09 mmol/L vs. 3.49 mmol/L, p = 0.002) than non-shift workers. HDL cholesterol, triglycerides, and HS-CRP levels did not significantly differ between shift and non-shift workers. After adjustment for covariates, differences in total and LDL cholesterol between shift and non-shift workers remained statistically significant (Table 2). Compared to non-shift workers, shift workers’ total cholesterol level was 0.38 mmol/L lower (95%-CI = -0.73 –-0.04, p = 0.030) and shift workers’ LDL cholesterol level was 0.34 mmol/L lower (95%-CI = -0.60 –-0.08, p = 0.011). For the anthropometrics and the other metabolic risk factors measured in blood, no significant differences between shift and non-shift workers were found.

**Table 2:** Differences in metabolic risk factors between shift workers and non-shift workers.

	Shift workers	Non-shift workers	Shift workers vs. non-shift workers†
	*Mean (SD); median*	*Mean (SD); median*	*B (95%-CI)*
BMI (in kg/m^2^) at T = 0^1^	25.3 (4.2); 24.7	25.3 (4.3); 24.9	0.40 (-0.61–1.41)
Waist circumference (in cm) at T = 0^1^	85.2 (11.4); 84.0	86.4 (12.3); 85.9	0.56 (-2.05–3.16)
Total cholesterol (in mmol/L)^2^	5.54* (1.12); 5.39	5.99* (1.26); 5.96	-0.38 (-0.73 –-0.04)*
HDL cholesterol (in mmol/L)^2^	1.86 (0.46); 1.79	1.86 (0.51); 1.75	-0.01 (-0.16–0.13)
LDL cholesterol (in mmol/L)^2^	3.09* (0.87); 3.03	3.49* (0.88); 3.54	-0.34 (-0.60 –-0.08)*
Triglycerides (in mmol/L)^2^	1.37 (0.77); 1.16	1.33 (0.87); 1.14	1.01 (0.87–1.17)^3^
HS-CRP (in mg/L)^2^	1.70 (1.88); 1.00	1.35 (1.35); 0.85	1.06 (0.77–1.47)^3^

B, regression coefficient; CI, confidence interval; HDL, high-density lipoprotein; HS-CRP, high-sensitivity C-reactive protein; LDL, low-density lipoprotein.

^†^ Adjusted for age, gender, occupation, educational level, general perceived health, smoking, and alcohol intake.

^1^ Baseline measurement (n = 596),

^2^ Follow-up measurement (n = 329, and n = 324 for HS-CRP)

^3^ Ratio between geometric means of shift workers and non-shift workers is shown for the regression coefficients of triglycerides and high-sensitivity C-reactive protein.

*p<0.05.

### Frequency and duration of night shift work and metabolic risk factors

Regarding frequency of night shift work, Table 3 shows that the LDL cholesterol levels of shift workers with 1–2 (B = -0.39 (95%-CI = -0.72 –-0.05), p = 0.025), 3–4 (B = -0.32 (95%-CI = -0.61 –-0.03), 0.032), and ≥5 night shifts/month (B = -0.32 (95%-CI = -0.63 –-0.01), p = 0.042) were all significantly lower than that of non-shift workers. Furthermore, total cholesterol and LDL cholesterol levels of shift workers working night shifts for ≥20 years were significantly lower than those of non-shift workers (total cholesterol: B = -0.61 (95%-CI = -1.00 –-0.23), p = 0.002, LDL: B = -0.49 (95%-CI = -0.78 –-0.19), p = 0.001). However, this was not found for shift workers working night shifts for <10 years (total cholesterol: B = -0.03 (95%-CI = -0.49–0.43), p = 0.890, LDL: B = -0.17 (95%-CI = -0.52–0.18), p = 0.335) and 10–19 years (total cholesterol: B = -0.19 (95%-CI = -0.61–0.23), p = 0.370, LDL: B = -0.18 (95%-CI = -0.50–0.14), p = 0.261). No associations between frequency and duration of night shift work and the other metabolic risk factors were found.

**Table 3:** Effect estimates of the differences in metabolic risk factors by frequency of night shifts and by duration of night shift work, compared to non-shift workers†.

	Non-night shift workers (n = 36) *B (95%-CI)*	1–2 night shifts/month (n = 79) *B (95%-CI)*	3–4 night shifts/month (n = 224) *B (95%-CI)*	≥5 night shifts/month (n = 164) *B (95%-CI)*	Non-night shift workers (n = 36) *B (95%-CI)*	<10 years (n = 174) *B (95%-CI)*	10–19 years (n = 114) *B (95%-CI)*	≥20 years (n = 179) *B (95%-CI)*
BMI (in kg/m^2^)	0.98 (-0.64–2.61)	-0.68 (-1.93–0.58)	0.69 (-0.41–1.80)	0.82 (-0.35–1.99)	0.85 (-0.78–2.48)	0.51 (-0.82–1.83)	0.77 (-0.45–2.00)	0.05 (-1.10–1.21)
Waist circumference (in cm)	2.18 (-2.03–6.38)	-1.41 (-4.66–1.84)	1.33 (-1.54–4.19)	0.74 (-2.28–3.77)	1.93 (-2.27–6.14)	0.58 (-2.82–3.99)	1.67 (-1.49–4.82)	-0.34 (-3.31–2.63)
Total cholesterol (in mmol/L)	-0.53 (-1.17–0.11)	-0.49* (-0.93 –-0.05)	-0.33 (-0.71–0.05)	-0.32 (-0.73–0.09)	-0.55 (-1.19–0.08)	-0.03 (-0.49–0.43)	-0.19 (-0.61–0.23)	-0.61* (-1.00 –-0.23)
HDL cholesterol (in mmol/L)	0.03 (-0.23–0.30)	-0.08 (-0.27–0.10)	-0.01 (-0.17–0.15)	0.03 (-0.14–0.20)	0.03 (-0.24–0.29)	0.14 (-0.05–0.33)	-0.02 (-0.20–0.15)	-0.09 (-0.25–0.08)
LDL cholesterol (in mmol/L)	-0.41 (-0.90–0.08)	-0.39* (-0.72 –-0.05)	-0.32* (-0.61 –-0.03)	-0.32* (-0.63 –-0.01)	-0.43 (-0.91–0.06)	-0.17 (-0.52–0.18)	-0.18 (-0.50–0.14)	-0.49* (-0.78 –-0.19)
Triglycerides (in ln of mmol/L)	0.84 (0.64–1.09)	1.02 (0.85–1.23)	1.02 (0.87–1.20)	1.02 (0.86–1.21)	0.83 (0.64–1.09)	1.03 (0.84–1.25)	1.03 (0.86–1.23)	1.02 (0.86–1.20)
HS-CRP (in ln of mg/L)	0.98 (0.54–1.79)	0.96 (0.64–1.46)	1.20 (0.84–1.71)	1.00 (0.68–1.46)	0.96 (0.53–1.74)	1.07 (0.69–1.65)	1.33 (0.89–1.97)	0.97 (0.67–1.40)

Reference group: non-shift workers

B, regression coefficient; CI, confidence interval; HDL, high-density lipoprotein; HS-CRP, high-sensitivity C-reactive protein; LDL, low-density lipoprotein; ln, natural logarithm.

^†^ Adjusted for age, gender, occupation, educational level, general perceived health, smoking, and alcohol intake.

Ratio between geometric means of shift workers and non-shift workers are shown for triglycerides and high-sensitivity C-reactive protein.

For blood parameters, corresponding n-values were non-night shift workers n = 15, 1–2 night shifts/month n = 44, 3–4 night shifts/month n = 125, ≥5 night shifts/month n = 88, <10 years n = 86, 10–19 years n = 63, ≥20 years n = 108.

*p<0.05.

### Chronotype of shift workers and metabolic risk factors

Table 4 shows the effect estimates of shift workers with different chronotypes compared to non-shift workers. Compared to non-shift workers, effect estimates for the different metabolic risk factors did not differ between shift workers with morning, evening, or intermediate chronotypes. For example, shift workers with morning (B = -0.37 (95%-CI = -0.66 –-0.08), p = 0.012), evening (B = -0.29 (95%-CI = -0.58 –-0.00), p = 0.048), and intermediate (B = -0.37 (95%-CI = -0.69 –-0.05), p = 0.023) chronotypes all had similar effect estimates for lower LDL cholesterol levels compared to non-shift workers.

**Table 4:** Effect estimates of the differences in metabolic risk factors by chronotype of shift workers compared to non-shift workers†.

	Shift workers with morning chronotype (n = 180) *B (95%-CI)*	Shift workers with evening chronotype (n = 207) *B (95%-CI)*	Shift workers with intermediate chronotype (n = 116) *B (95%-CI)*
BMI (in kg/m^2^)	0.12 (-0.99–1.22)	0.70 (-0.43–1.82)	0.47 (-0.74–1.67)
Waist circumference (in cm)	-0.24 (-3.08–2.60)	2.01 (-0.87–4.89)	-0.19 (-3.29–2.91)
Total cholesterol (in mmol/L)	-0.39* (-0.77 –-0.01)	-0.36 (-0.74–0.03)	-0.41 (-0.84–0.01)
HDL cholesterol (in mmol/L)	0.02 (-0.13–0.18)	-0.06 (-0.22–0.10)	0.00 (-0.17–0.18)
LDL cholesterol (in mmol/L)	-0.37* (-0.66 –-0.08)	-0.29* (-0.58 –-0.00)	-0.37* (-0.69 –-0.05)
Triglycerides (in ln of mmol/L)	0.96 (0.81–1.13)	1.03 (0.87–1.21)	1.07 (0.89–1.27)
HS-CRP (in ln of mg/L)	0.95 (0.67–1.36)	1.15 (0.80–1.65)	1.13 (0.76–1.68)

Reference group: non-shift workers

B, regression coefficient; CI, confidence interval; HDL, high-density lipoprotein; HS-CRP, high-sensitivity C-reactive protein; LDL, low-density lipoprotein; ln, natural logarithm.

^†^ Adjusted for age, gender, occupation, educational level, general perceived health, smoking, and alcohol intake.

Ratio between geometric means of shift workers and non-shift workers are shown for triglycerides and high-sensitivity C-reactive protein.

For blood parameters, corresponding n-values were shift workers with morning chronotype n = 96, shift workers with evening chronotype n = 113, shift workers with intermediate chronotype n = 63.

*p<0.05.

## Discussion

In this study among health care workers employed in hospitals, shift workers’ total and LDL cholesterol levels were lower than those of non-shift workers, but no differences between shift and non-shift workers were found in weight-related measures (BMI and waist circumference), HDL cholesterol, triglycerides, and HS-CRP. The results were similar for shift workers working different numbers of night shifts/month. Stratified analyses by duration of night shift work showed lower LDL cholesterol levels for shift workers working night shifts for ≥20 years, while this association was not found for shift workers working night shifts for <10 years and 10–19 years. Chronotype of shift workers did not appear to be associated with differences in any of the metabolic risk factors.

Previous review studies found evidence for an effect of shift work on body weight [3, 4, 13], while the current study did not find a difference between shift and non-shift workers in weight-related measures. A possible explanation for this may be that the reviews were based on studies among different study populations, with different occupations and demographics. For example, the high-quality studies that found a positive association between shift work and weight-related measures in the review of Proper et al. (2016) all had populations consisting of male, blue-collar workers.[3] The results may therefore not be completely generalizable to the current study that was performed on, mostly female, health care workers. Similarly, Van Drongelen et al. (2011) concluded that the all-male, high-quality studies that were analyzed found an adjusted association between shift work and weight gain, but that this was not found in studies of female nurses.[4] The recent review of Sun et al. (2018) did report an increased risk of overweight/obesity among health care workers, but also indicated that they found a high degree of heterogeneity and that studies with more accurate measurements of night shift work and obesity should be conducted.[27]

For the association between shift work and blood lipids, previous review studies have found less support.[3, 13, 28] In general, it has been concluded that there is insufficient evidence for an association or no clear association between shift work and cholesterol.[3, 13] In accordance with these conclusions, no clear association between shift work and HDL cholesterol and triglycerides was found in the current study. However, total and LDL cholesterol levels of shift workers were lower than those of non-shift workers, although differences in total cholesterol can be explained by the differences in LDL cholesterol. As it was expected that shift workers would have a less optimal lipid profile than non-shift workers [28], this finding appears to be somewhat surprising. This finding cannot be explained by the total study population being relatively healthy or unhealthy, because the study participants had levels of the metabolic risk factors that appear to be representative for the general Dutch (working) population.[29] One possible explanation for the lower levels of LDL cholesterol in shift workers might be a selection effect within the total study population: non-shift workers may in general represent a less healthy group than shift workers. It may be that non-shift workers decided not to start shift work at all or stopped doing shift work due to health reasons. Therefore, the lower LDL cholesterol levels in shift workers could represent a healthy worker effect. The finding that especially the shift workers performing night shifts for ≥20 years had lower LDL cholesterol than non-shift workers may also point to a selection effect of “healthier” shift workers in the group working the longest in night shift work. Similarly, two previous studies reported more favorable lipid profiles among female health care workers with a longer shift work duration.[30, 31] Both studies related these findings to the healthy worker effect, indicating that workers who are best able to adapt to the shift work and who have a better general health status are most likely to continue shift work.[30, 31]

When translating the total cholesterol levels to a dichotomous measure of hypercholesterolemia (total cholesterol ≥6.5 mmol/L vs. <6.5 mmol/L), shift workers had a significantly lower odds of hypercholesterolemia than non-shift workers (OR = 0.46, 95%-CI = 0.22–0.97). Furthermore, LDL cholesterol reduction has been found to have a continuous relation with risk reduction of disease and vascular events, irrespective of baseline cholesterol concentration.[32, 33] Therefore, the observed difference in LDL cholesterol between shift and non-shift workers may be related to clinically relevant outcomes, such as a decreased risk of coronary heart disease and stroke.[32, 33] Nonetheless, as previous research has indicated that small dense LDL particles are more atherogenic than larger LDL particles [34, 35], more research is needed to compare LDL phenotypes of shift and non-shift workers also taking into account different subgroups of LDL cholesterol (e.g. very-low-density lipoprotein).

Even in individuals with normal LDL cholesterol levels, a high level of HS-CRP, which is a marker of inflammation, has been shown to be an important predictor for cardiovascular events.[11, 12] Compared to the other metabolic risk factors, less research has been done into the association between shift work and HS-CRP.[13] Some studies have reported that shift work may increase HS-CRP levels [36–38], but, in line with the current study, others did not find an association between shift work and HS-CRP.[39, 40] Furthermore, one of these latter studies also indicated that HS-CRP levels neither correlated with number of night shifts nor with duration of night shift work, as was found in the current study.[39] Further research on this topic, including more research among female non-blue-collar workers, is necessary.

In our study to the role of chronotype, it was hypothesized that morning type shift workers would experience greater disruption of metabolic risk factors compared to evening types, because they may be less able to adapt to shift work.[1, 14, 15] However, we found no differences between shift workers with morning, evening, and intermediate chronotypes. Nonetheless, as morning types in general have been found to have a lower risk for cardiovascular diseases and diabetes than evening types [41, 42], more research is needed to study whether the potential negative effect of shift work on cardio-metabolic outcomes for morning types could to some extent be counteracted by the general “protective” effects of their morning chronotype.[43]

Main lifestyle behaviors such as diet, physical activity, and sleep were not included as covariates in this study, because they may play an important role in the causal pathway between shift work and metabolic risk factors. The descriptive information about these lifestyle behaviors presented in Table 1 indicates that physical activity at work and sleep quality differed between shift- and non-shift workers in this study population, while there were no significant differences in leisure-time physical activity, sleep duration, and meal and snack frequency. Although these measures are based on self-reported data and may therefore be prone to bias (e.g. overestimation of physical activity levels), they are useful in comparing lifestyle behaviors between shift- and non-shift workers. Furthermore, these results are consistent with objectively measured findings for physical activity and sleep in a subsample of the Klokwerk+ study population.[44, 45] If these lifestyle behaviors mediate the relation between shift work and metabolic risk factors, which has to be confirmed in longitudinal studies, then the observed differences in lifestyle behaviors in the current study population might not be large enough to result in differences in metabolic risk factors. The shift workers included in the current study are generally highly educated and could be aware of the fact that shift work may be associated with negative health outcomes. Therefore, they may attempt to compensate for this by trying to adopt a healthier lifestyle, resulting in smaller differences compared to their non-shift working colleagues than one might expect.

### Strengths and limitations

The strengths of this study were the use of detailed information on shift work status and objective instead of self-reported measures of anthropometrics as well as the other metabolic risk factors. Furthermore, the chronotype of shift workers and possible confounding factors were taken into account.

Some limitations should also be noted. Due to the sample size of the group of non-shift workers, it was not possible to perform more detailed analyses within this group, such as analyses stratified for chronotype. The sample of non-shift workers may also be too small to completely determine differences in metabolic risk factors between shift- and non-shift workers. Furthermore, only one cross-sectional measurement of metabolic risk factors was used in this study. However, the focus of the current study was not on determining direct effects of shift work, for which multiple measurements would be required, but on the association between chronic, more long-term exposure to shift work and metabolic risk factors. Therefore, all shift workers had a history of shift work of more than six months before enrolling in the study. Due to practical considerations, participants were not instructed to fast before blood sample collection. Conventionally, it is common practice to measure the lipid profile in blood obtained after fasting for at least 8 hours.[46] However, a recent study has shown that differences between fasting and non-fasting lipid profiles are not clinically significant, and the authors have recommended that non-fasting blood samples can be routinely used for the assessment of lipid profiles.[46] All blood samples were collected in the morning, and further analysis revealed that specific timing of blood sample collection did not affect results. Furthermore, taking into account dichotomous cut-off points for metabolic risk factors (unhealthy vs. healthy values) instead of continuous measures, shift work history for the non-shift working group, and whether shift workers recently worked night shifts (yesterday or in the last three days), all did not affect the conclusions of the current study. Lastly, the results of the current study apply to shift workers employed in the health care sector, and cannot be directly translated to other populations of shift workers. By recruiting shift and non-shift workers from the same work environments (i.e. the same hospitals), comparability between these groups of health care workers was increased. Nonetheless, as shown in Table 1, shift and non-shift workers still differed in for example educational level and occupation. Although the results were adjusted for these variables, residual confounding due to potential differences in other (work-related) characteristics cannot be ruled out.

## Conclusions

In conclusion, metabolic risk factors did not differ between shift and non-shift workers, except that shift workers had lower levels of total and LDL cholesterol than non-shift workers. This association between shift work and LDL cholesterol was found especially among shift workers working night shifts for ≥20 years, but not among shift workers working night shifts for <10 years and 10–19 years, which may relate to a healthy worker selection effect. No differences were found for shift workers working different numbers of night shifts/month and for shift workers with different chronotypes. These results do not support an underlying mechanistic role of the studied metabolic risk factors as a potential link between shift work and cardio-metabolic health effects in this population of health care workers. Further research that takes into account different exposure aspects of shift work and individual characteristics of shift workers is recommended to establish a better understanding of the association between shift work, metabolic risk factors, and cardio-metabolic diseases.

## Supporting information

S1 File Copy of the survey questions used in the study in the original language (Dutch). (DOCX)

S2 File Copy of the survey questions used in the study in English. (DOCX)
