# Supplementary material for: The association between exposure to different aspects of shift work and metabolic risk factors in health care workers, and the role of chronotype
Source: PLoS One. 2019 Feb 1;14(2):e0211557. doi: 10.1371/journal.pone.0211557 (PMC6358078; doi:10.1371/journal.pone.0211557)
Supplement: S1 File — (DOCX) [file pone.0211557.s001.docx]

**S1. Copy of the survey questions used in the manuscript “The association between exposure to different aspects of shift work and metabolic risk factors in health care workers, and the role of chronotype” in the original language (Dutch).**

**1. ALGEMEEN**

1.1 Wat is uw geboortejaar?

19

1.2 Wat is uw geslacht?

- Man
- Vrouw

1.3 In welk land bent u geboren?

- Nederland
- Anders, namelijk:

1.4 Wat is uw huidige burgerlijke staat?

- Gehuwd / geregistreerd partnerschap / samenwonend
- Gescheiden of gescheiden levend
- Weduwe
- Relatie maar niet samenwonend (LAT-relatie)
- Alleenstaand (geen partner)
- Inwonend bij ouder(s)

1.5 Wat is uw hoogst genoten opleiding?

- Basisschool
- Mavo/VMBO
- Havo/vwo
- MBO niveau 1 t/m 3
- MBO niveau 4
- HBO
- WO

1.6 Wat is uw beroep?

- Arts
- Verpleegkundige
- Paramedicus (bijv. diëtist, fysiotherapeut, ergotherapeut)
- Verzorgende
- Anders, namelijk:

1.7 Wat vindt u, over het algemeen genomen, van uw gezondheid?

- Uitstekend
- Zeer goed
- Goed
- Matig
- Slecht

**2. NACHTDIENSTEN EN ONREGELMATIGE DIENSTEN**

2.1a Vul hieronder voor elk type dienst die voor u van toepassing is in uw huidige baan de normale werktijden in en het aantal diensten dat u gemiddeld per maand van elk type draait:

| Dienst | Begintijd  bijv. 23:00 | Eindtijd  bijv. 07:00 | Aantal diensten per maand | Niet van toepassing |
| --- | --- | --- | --- | --- |
| Ochtend |  |  |  |  |
| Dag |  |  |  |  |
| Avond |  |  |  |  |
| Nacht |  |  |  |  |
| Slaap |  |  |  |  |
| Anders, namelijk: |  |  |  |  |

2.2a Heeft u ooit onregelmatige diensten gedraaid (dit zijn diensten waarin dag-, nacht-, avond- en/of slaapdiensten elkaar afwisselen)?

- Ja
- Nee

2.2b Hoeveel jaar/maanden heeft u in totaal onregelmatige diensten gedraaid?

jaar en maand(en)

2.2c Wanneer bent u hiermee begonnen?

- - (dag – maand - jaar)

2.2d Draait u nog steeds onregelmatige diensten?

- Ja
- Nee

2.2e Wanneer bent u hiermee gestopt?

- - (dag – maand – jaar)

2.3a Heeft u ooit nachtdiensten gedraaid?

- Ja
- Nee

2.3b Hoeveel jaar/maanden heeft u in totaal nachtdiensten gedraaid?

jaar en maand(en)

2.3c Wanneer bent u hiermee begonnen?

- - (dag – maand – jaar)

2.3d Draait u nog steeds nachtdiensten?

- Ja
- Nee

2.3e Wanneer bent u hiermee gestopt?

- - (dag – maand – jaar)

2.3f Hoeveel nachtdiensten draaide u gemiddeld per maand in deze periode?

(aantal nachtdiensten)

**3. CHRONOTYPE EN FLEXIBILITEIT**

3.1 Als welk type zou u zichzelf omschrijven?

- Duidelijk meer een ochtendmens
- Meer ochtend dan avondmens
- Meer avond dan ochtendmens
- Duidelijk meer een avondmens
- Geen specifiek type/flexible

**4. SLAAP**

4.1 Hoe zou u uw slaapkwaliteit in de afgelopen 4 weken over het algemeen beoordelen?

- Zeer goed
- Redelijk goed
- Redelijk slecht
- Zeer slecht

4.2 Hoeveel uur sliep u gemiddeld tijdens uw voornaamste slaapperiode van de dag in de afgelopen 4 weken?

uur en minuten

**5. BEWEGEN**

*Neem in uw gedachten een normale week in de afgelopen maand. Wilt u aangeven hoeveel dagen per week u de onderstaande activiteiten verrichtte en hoeveel tijd u daar gemiddeld op zo'n dag mee bezig was.*

5.1 Woon/werkverkeer.

| *Als u een activiteit niet heeft verricht, vul dan een 0 in.* | Aantal dagen per week | Gemiddelde tijd per dag |
| --- | --- | --- |
| a. Lopen van/naar werk | ... | ... uur ... min |
| b. Fietsen van/naar werk | ... | ... uur ... min |

5.2 Lichamelijke activiteit op werk.

| *Als u een activiteit niet heeft verricht, vul dan een 0 in.* | Aantal uren per week |
| --- | --- |
| a. Licht en matig inspannend werk (zittend, staand werk met af en toe lopen, zoals bureau werk of lopend werk met lichte lasten). | ... uur |
| b. Zwaar inspannend werk (lopend werk of werk waarbij regelmatig zware dingen moeten worden opgetild). | ... uur |

5.3 Huishoudelijke activiteiten.

| *Als u een activiteit niet heeft verricht, vul dan een 0 in.* | Aantal dagen per week | Gemiddelde tijd per dag |
| --- | --- | --- |
| a. Licht en matig inspannend werk (staand werk, zoals koken, afwassen, strijken, kind eten geven/ in bad doen en lopend werk zoals stofzuigen, boodschappen doen ). | ... | ... uur ... min |
| b. Zwaar inspannend werk (zoals vloeren schrobben, tapijt uitkloppen, met zware boodschappen lopen). | ... | ... uur ... min |

5.4 Vrije tijd.

| *Als u een activiteit niet heeft verricht, vul dan een 0 in.* | Aantal dagen per week | Gemiddelde tijd per dag |
| --- | --- | --- |
| a. Wandelen | ... | ... uur ... min |
| b. Fietsen | ... | ... uur ... min |
| c. Tuinieren | ... | ... uur ... min |
| d. Klussen/doe-het-zelven | ... | ... uur ... min |

5.5 Sport.

*(Hier maximaal 4 sporten opschrijven bijv. fitness, tennis, trimmen/joggen, voetbal).*

|  | Aantal dagen per week | Gemiddelde tijd per dag |
| --- | --- | --- |
| …………………………………………………………………….. | ... | ... uur ... min |
| …………………………………………………………………….. | ... | ... uur ... min |
| …………………………………………………………………….. | ... | ... uur ... min |
| …………………………………………………………………….. | ... | ... uur ... min |

**6. ALCOHOL & ROKEN**

6.1a Op hoeveel dagen van de maandag t/m donderdag drinkt u gemiddeld genomen alcoholhoudende drank?

- 4 dagen
- 3 dagen
- 2 dagen
- 1 dag
- Minder dan 1 dag
- Ik drink nooit op maandag t/m donderdag

6.1b Hoeveel glazen drinkt u dan gemiddeld op zo’n door-de-weekse dag?

- 11 of meer glazen
- 7 - 10 glazen
- 6 glazen
- 5 glazen
- 4 glazen
- 3 glazen
- 2 glazen
- 1 glas

6.2a Op hoeveel dagen van de vrijdag t/m zondag drinkt u gemiddeld genomen alcoholhoudende drank?

- 3 dagen
- 2 dagen
- 1 dag
- Minder dan 1 dag
- Ik drink nooit op vrijdag t/m zondag

6.2b Hoeveel glazen drinkt u dan gemiddeld op zo’n weekend-dag?

- 11 of meer glazen
- 7 - 10 glazen
- 6 glazen
- 5 glazen
- 4 glazen
- 3 glazen
- 2 glazen
- 1 glas

6.3 Rookt u wel eens?

- Ja
- Nee
