# Supplementary material for: The association between exposure to different aspects of shift work and metabolic risk factors in health care workers, and the role of chronotype
Source: PLoS One. 2019 Feb 1;14(2):e0211557. doi: 10.1371/journal.pone.0211557 (PMC6358078; doi:10.1371/journal.pone.0211557)
Supplement: S2 File — (DOCX) [file pone.0211557.s002.docx]

**S2. Copy of the survey questions used in the manuscript “The association between exposure to different aspects of shift work and metabolic risk factors in health care workers, and the role of chronotype” in English.**

**1. GENERAL**

1.1 What is your year of birth?

19

1.2 What is your gender?

- Male
- Female

1.3 In what country were you born?

- The Netherlands
- Other, namely:

1.4 What is your current marital status?

- Married / Living together
- Separated
- Widow(er)
- In a relationship, but not living together
- Single
- Living with parents

1.5 What is your highest completed level of education?

- Primary school
- Intermediate vocational education
- Higher secondary education
- Higher vocational education
- University

1.6 What is your occupation?

- Physician
- Nurse
- Paramedic (e.g. dietician, physiotherapist)
- Health care assistant
- Other, namely:

1.7 In general, would you say your health is

- Excellent
- Very good
- Good
- Fair
- Poor

**2. NIGHT SHIFTS AND ROTATING SHIFTS**

2.1a For each shift type that applies to you in your current job, please enter the normal working hours and the number of shifts you work on average per month:

| Shift | Start time  e.g. 23:00 | End time  e.g. 07:00 | Number of shifts per month | Not applicable |
| --- | --- | --- | --- | --- |
| Morning |  |  |  |  |
| Day |  |  |  |  |
| Evening |  |  |  |  |
| Night |  |  |  |  |
| Sleep |  |  |  |  |
| Other, namely: |  |  |  |  |

2.2a Have you ever worked rotating shifts (i.e. shifts rotating between day, evening, night and/or evening shifts)?

- Yes
- No

2.2b In total, how many years and months have you worked rotating shifts?

years and months

2.2c When did you start working rotating shifts?

- - (day – month - year)

2.2d Do you still work rotating shifts?

- Yes
- No

2.2e When did you stop working rotating shifts?

- - (day – month - year)

2.3a Have you ever worked night shifts?

- Yes
- No

2.3b In total, how many years and months have you worked rotating shifts?

years and months

2.3c When did you start working night shifts?

- - (day – month - year)

2.3d Do you still work night shifts?

- Yes
- No

2.3e When did you stop working night shifts?

- - (day – month - year)

2.3f How many night shifts did you work on average each month during this time?

(number of night shifts)

**3. CHRONOTYPE**

3.1 As which type would you describe yourself?

- Definitely a morning type
- More a morning type than an evening type
- More an evening type than a morning type
- Definitely an evening type
- No specific type/intermediate type

**4. SLEEP**

4.1 During the past 4 weeks, how would you rate your sleep quality overall?

- Very good
- Fairly good
- Fairly bad
- Very bad

4.2 On the average, how many hours did you sleep during your main sleep period of the day during the past 4 weeks?

hours and minutes

**5. Physical activity**

*Think about an average week in the past months. Please indicate how many days per week you performed the following activities, and how much time on average you were engaged in this.*

5.1 Commuting activities.

| *If you did not perform an activity, please enter a 0.* | Days per week | Average time per day |
| --- | --- | --- |
| a. Walking to/from work | ... | hours minutes |
| b. Bicycling to/from work | ... | hours minutes |

5.2 Activity at work.

| *If you did not perform an activity, please enter a 0.* | Average time per day |
| --- | --- |
| a. Light work (sitting/standing with some walking, e.g., a desk job). | hours minutes |
| b. Intense work (walking or regularly lifting heavy objects at work). | hours minutes |

5.3 Household activities.

| *If you did not perform an activity, please enter a 0.* | Days per week | Average time per day |
| --- | --- | --- |
| a. Light household work (cooking, washing dishes, ironing, child care) | ... | hours minutes |
| b. Intense household work (scrubbing floor, walking with heavy shopping bags) | ... | hours minutes |

5.4 Leisure time activities.

| *If you did not perform an activity, please enter a 0.* | Days per week | Average time per day |
| --- | --- | --- |
| a. Walking | ... | hours minutes |
| b. Bicycling | ... | hours minutes |
| c. Gardening | ... | hours minutes |
| d. Odd jobs | ... | hours minutes |

5.5 Sports.

*(Please write down yourself, e.g. tennis, fitness, skating, swimming, dancing).*

|  | Days per week | Average time per day |
| --- | --- | --- |
| …………………………………………………………………….. | ... | hours minutes |
| …………………………………………………………………….. | ... | hours minutes |
| …………………………………………………………………….. | ... | hours minutes |
| …………………………………………………………………….. | ... | hours minutes |

**6. ALCOHOL & SMOKING**

6.1a On average, on how many days from Monday to Thursday do you drink alcoholic beverages?

- 4 days
- 3 days
- 2 days
- 1 day
- Less than 1 day
- I never drink alcohol beverages from Monday to Thursday

6.1b On average, how many glasses do you drink on such a weekday?

- 11 or more glasses
- 7 - 10 glasses
- 6 glasses
- 5 glasses
- 4 glasses
- 3 glasses
- 2 glasses
- 1 glass

6.2a On average, on how many days from Friday to Sunday do you drink alcoholic beverages?

- 3 days
- 2 days
- 1 day
- Less than 1 day
- I never drink alcohol beverages from Friday to Sunday

6.2b On average, how many glasses do you drink on such a weekend day?

- 11 or more glasses
- 7 - 10 glasses
- 6 glasses
- 5 glasses
- 4 glasses
- 3 glasses
- 2 glasses
- 1 glass

6.3 Do you sometimes smoke?

- Yes
- No
